# Dimeric and tetrameric forms of muscle fructose-1,6-bisphosphatase play different roles in the cell

**DOI:** 10.18632/oncotarget.23271

**Published:** 2017-12-15

**Authors:** Janusz Wiśniewski, Michał Piróg, Rafał Hołubowicz, Piotr Dobryszycki, James A. McCubrey, Dariusz Rakus, Agnieszka Gizak

**Affiliations:** ^1^ Department of Molecular Physiology and Neurobiology, University of Wroclaw, Wroclaw 50-335, Poland; ^2^ Department of Biochemistry, Wroclaw University of Science and Technology, Wroclaw 50-370, Poland; ^3^ Department of Microbiology and Immunology, Brody School of Medicine at East Carolina University, Greenville, NC, USA

**Keywords:** allostery, cardiomyocytes, mitochondria, ROS production, FBP

## Abstract

Muscle fructose 1,6-bisphosphatase (FBP2), besides being a regulatory enzyme of glyconeogenesis also protects mitochondria against calcium stress and plays a key role in regulation of the cell cycle, promoting cardiomyocytes survival. However, in cancer cells, FBP2 acts as an anti-oncogenic/anti-proliferative protein. Here, we show that the physiological function of FBP2 depends both on its level of expression in a cell as well as its oligomerization state. Animal fructose-1,6-bisphosphatases are thought to function as tetramers. We present evidence that FBP2 exists in an equilibrium between tetramers and dimers. The dimeric form is fully active and insensitive to AMP, the main allosteric inhibitor of FBP2. Tetramerization induces the sensitivity of the protein to AMP, but it requires the presence of a hydrophobic central region in which leucine 190 plays a crucial role. Only the tetrameric form of FBP2 is retained in cardiomyocyte cell nucleus whereas only the dimeric form associates with mitochondria and protects them against stress stimuli, such as elevated calcium and H_2_O_2_ level. Remarkably, in hypoxic conditions, which are typical for many cancers, FBP2 ceases to interact with mitochondria and loses its pro-survival potential. Our results throw new light on the basis of the diverse role of FBP2 in cells.

## INTRODUCTION

Fructose 1,6-bisphosphatase (FBP), a regulatory gluconeogenic enzyme, was shown to have functions far exceeding regulation of glucose/glycogen synthesis. It participates in cardiac mitochondria protection against calcium stress [1–2]. It also plays a role in regulation of the cell cycle – it is transported into the nucleus in the late G1 phase and accumulates there through the S and G2 phases both in cardiomyocytes and in cancer cells. Nuclear FBP appears to promote cell survival, as withdrawal of the protein from the nucleus correlates with increased mortality of cardiomyocytes [3–7]. On the other hand, it was also shown that FBP acts as an anti-oncogenic protein both by its enzymatic, “anti-glycolytic” activity and by an interaction with HIF-1, a master glycolysis activator and that in some cancers, FBP is depleted [8–13].

While the pro-proliferative and stress-protective action of FBP is associated with the muscle isoform (FBP2), the anti-oncogenic function seems to be common for both the isoforms: FBP2 and FBP1 (the liver isoform). Thus, it seems that FBP promotes both survival and mortality of a cell. Factors determining which of these two contradictory effects are dominant remain unknown and this apparent paradox remains unresolved.

In the present paper, we present evidence that the pro-survival role of FBP2 is dependent on the enzyme’s quaternary structure.

Mammalian FBP has been previously described as a solely tetrameric protein, composed of two identical dimers, so called upper and lower dimer. Depending on a relative orientation of the dimers, the tetramer can exist in an active R-state or an inactive T-state. The switch between R- and T-state is caused by binding of AMP, the enzyme’s most potent allosteric inhibitor [14–21].

Here, we show that in the absence of allosteric effectors (AMP and NAD^+^), only about half of the total FBP2 species are tetramers and significant amounts of dimers and monomers exist in the solution. Addition of AMP induces almost complete tetramerization of the wild-type FBP2 (WT FBP2) even at submicromolar AMP concentrations. A dimer-tetramer equilibrium has never been demonstrated before for any of the two mammalian isoforms of FBP nor was the presence of free monomers shown for any FBP.

Crystal structures of human muscle FBP2 in the R- and T-states presented in our previous study highlighted residues D187 and L190 as potentially necessary for existence of the R-state tetramer [21]. Here, we show that the tetramerization is disrupted partially by D187L and completely by L190G substitution. Such recombinant dimeric FBP2 forms retain catalytic activity. However, their sensitivity to AMP is decreased and in contrast to the WT FBP2, the mutants oligomerization state is unaffected (L190G) or only slightly affected (D187L) by increasing AMP concentrations. The classical model of FBP2 inhibition by AMP assumes that binding of AMP to the allosteric site of one subunit in an FBP dimer stabilizes the so-called catalytic loop belonging to a subunit in the adjacent dimer in its inactive conformation [22–23]. Results of our newest kinetic experiments suggest that in high concentrations of AMP, there is an alternative mechanism of FBP2 inhibition by this compound.

In our previous papers, we have shown that the quaternary structure of FBP2 influences not only its enzymatic activity, but also the ability to bind to mitochondria and that saturation of FBP2 with AMP prevents binding of the enzyme to these organelles [2]. We have also characterized physiological factors required to stimulate nuclear transport of FBP2 [6] and identified the functional Nuclear Localization Sequence (NLS) within the FBP2 structure [5].

Here, we show that both nuclear and mitochondrial roles of FBP2 are strictly related to its oligomerization state. We show that only the dimeric FBP2 can interact with mitochondria and protect them against stress stimuli, however, unexpectedly, FBP2 loses its pro-survival potential in hypoxic conditions. On the other hand, only the tetrameric form is retained in the nuclei. This striking difference of function between two oligomeric forms of the same enzyme offers new, exciting possibilities. It can be envisioned that modulation of the oligomerization state of FBP2 could enable switching its action from promotion of cell survival to induction of death.

## RESULTS & DISCUSSION

### FBP2 promotes cell survival under oxidative stress but impairs it under hypoxic conditions

Previously, we have shown that in HL-1 cells with down-regulated FBP2 (the FBP2- cells), the mitochondria are more susceptible to stress-induced depolarization [1]. Subsequently, we constructed the HL-1 cell line with overexpression of FBP2 (the FBP2+ cells). PCR analysis demonstrated that the ratio of FBP2 mRNA in the HL-1 FBP2-, wild-type (WT) and FBP2+ cells was 0.28 : 1 : 1.71. More importantly, these differences were also visible at the protein level (see Supplementary Figure 1). Using the MTT assay we found that under control conditions, the viability of the FBP2+ cells was about 1.4-time higher than WT (p=0.02) and about 2-times higher than FBP2- cells (p<0.001).

**Figure 1:**
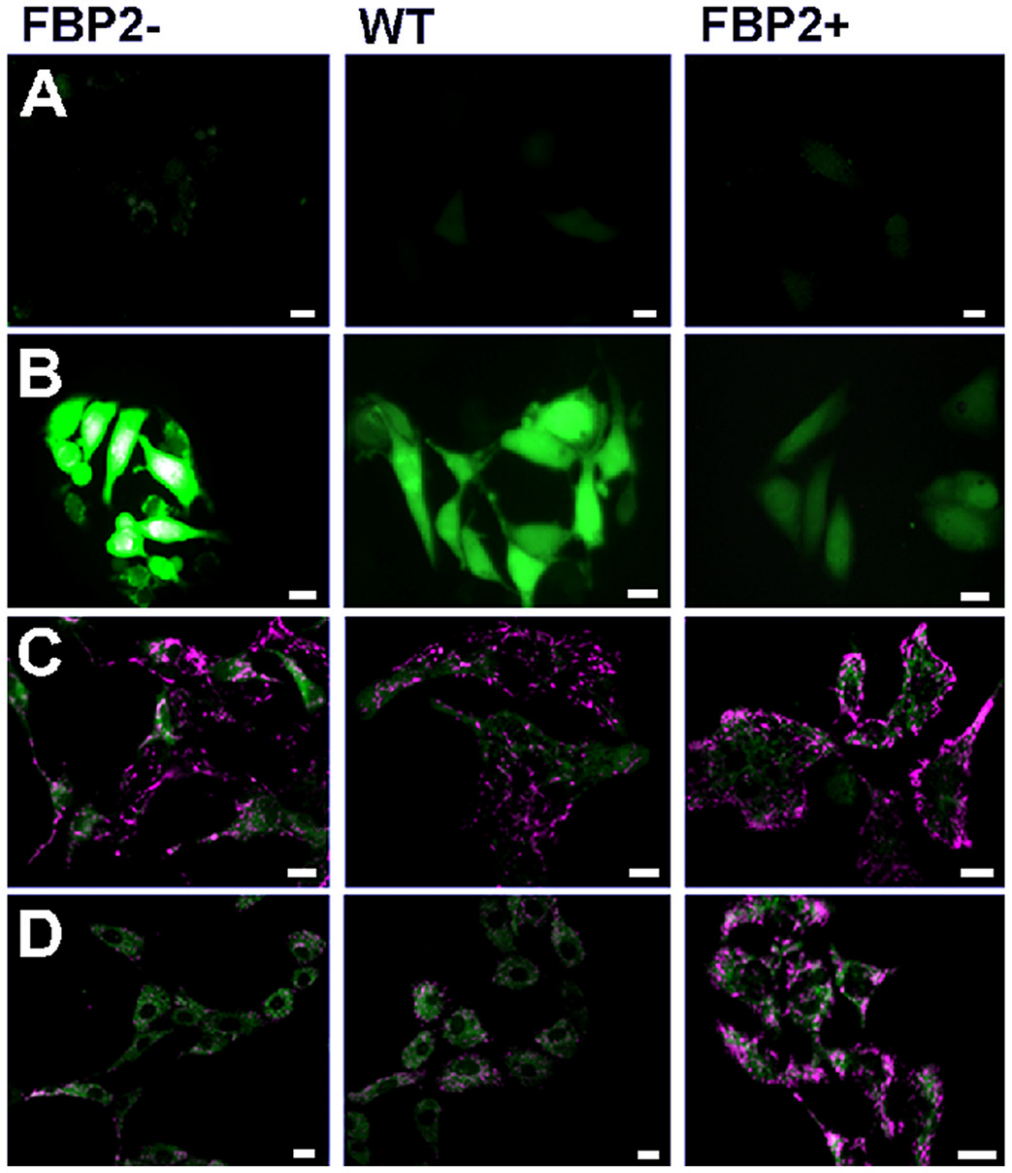
Comparison of the sensitivity of HL-1 cells with different levels of FBP2 expression to oxidative stress (H2O2) **(A, B)**. Production of ROS in control conditions (A) and under H_2_O_2_ (B) – dihydrofluorescein diacetate staining; **(C, D)**. Polarization of mitochondrial network in control conditions (C) and under H_2_O_2_ (D) – JC-1 staining. Bar = 10 μm.

Then, we compared cellular ROS production and mitochondrial polarization under stress induced by hyperoxic conditions (H_2_O_2_) in cells with the different levels of FBP2 expression. It turned out that cellular ROS production was inversely proportional while mitochondrial polarization was directly proportional to the level of FBP2 expression (Figure 1). Results of the MTT assay revealed that under the H_2_O_2_ treatment the FBP2+ cells were, respectively, almost 1.9- and 3.7-times more viable than WT and FBP2- cells. Importantly our pilot study on the mouse lung squamous cell carcinoma line (KLN-205) demonstrated that such protection against ROS might be a common mechanism as the KLN-205 cells overexpressing FBP2 were, respectively, about 1.25- and 3.1-times more viable than WT and FBP2- KLN-205 cells (see Supplementary Figure 2). Thereafter, we checked viability of the HL-1 cells under hypoxic conditions. In such conditions, we observed a reverse trend and now the FBP2- cells were about 1.7- and 4-times more viable (p<0.05) than WT and FBP2+ cells, respectively. Taken together, these results show that FBP2 overexpression increases the HL-1 cells growth and/or survival in normoxia and under hyperoxic conditions but impairs it in hypoxia.

### D187L and L190G mutations disrupt AMP inhibition of FBP2

**Figure 2:**
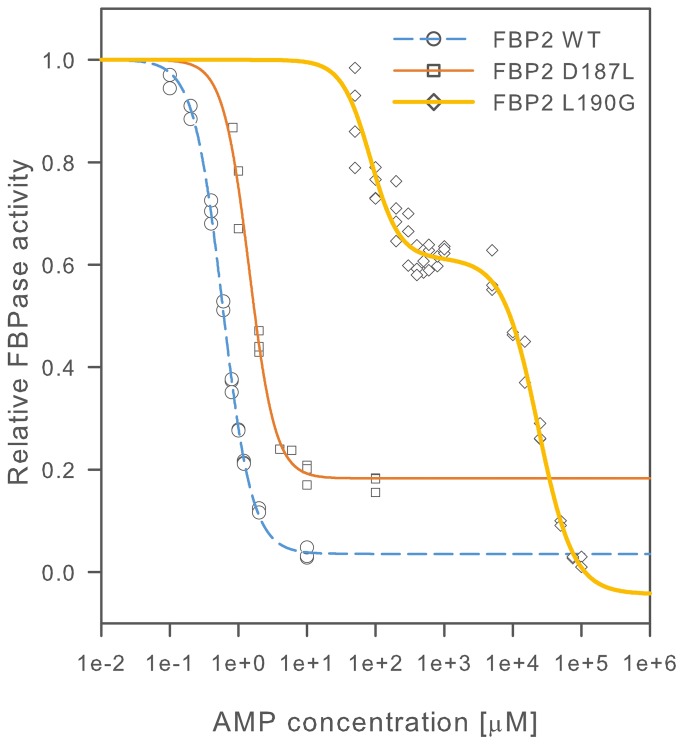
AMP inhibition of wild-type and mutant FBP2 Activity is presented as a fraction of FBPase activity in the absence of inhibitor. Curves were fitted to experimental data points as described in the materials and methods section. At least 3 independent measurements were performed for each AMP concentration.

To resolve the FBP2 structure-function relationship we substituted residues D187 and L190, which were indicated by crystallographic studies as potentially important for proper quaternary structure of FBP2 [21], with leucine and glycine residues, respectively. All the FBP2 forms were purified to homogeneity (purity >95%). We then investigated a general biochemical characteristics of the mutants, their interaction with cell nuclei and mitochondria, and their role in regulation of cell survival. As AMP is the most important regulator of FBP2 quaternary structure [21, 24] we looked for differences in its effect on the kinetics of the WT FBP2 and the mutants. To that end, we first established that the D187L and L190G mutants retained enzymatic activity similar to the WT FBP, although they required, three- and two-times higher Mg^2+^ concentration for maximal activity, respectively. Table 1 summarizes kinetic parameters of fructose-1,6-bisphosphate (F-1,6-BP) hydrolysis reaction, activation by Mg^2+^ and inhibition by AMP for D187L and L190G mutants compared with the WT FBP2 and WT FBP1. Figure 2 shows a comparison of AMP inhibition curves for WT, D187L and L190G FBP2.

**Table 1:** Kinetic parameters of mutant and wild-type forms of FBP1 and FBP2

	k_cat_ [s^−1^] ^a^	k_s_[μM], F-1,6-BP ^a^	k_is_[μM], F-1,6-BP ^a^	n, F-1,6-BP ^a^	A_50_[mM], Mg^2+^	n, Mg^2+^	IC_50_, AMP [μM] ^a^	n, AMP ^a^
FBP2 WT	20.9±2.52	3.66±0.07	111±2.3	1.61±0.04	0.88±0.05	1.96±0.21	0.59±0.01	2.04±0.05
FBP2 D187L	15.3±0.56	12.9±0.64	120±12	1.72±0.05	3.22±0.23	2.05±0.27	1.44±0.06	2.29±0.21
FBP2 L190G	23.8±3.19	5.82±0.20	119±3.6	1.99±0.06	1.96±0.09	1.94±0.17	84.4±6.7323300±2520	2.06±0.331.68±0.25
FBP1 ^b^	16.7±0.5	2.2±0.4	157±48	1	0.31±0.01	1.93±0.12	5.06±0.27	1.95±0.16

Data presented as mean±SD

^a^ – determined at Mg^2+^ concentration of 5 mM for wild-type FBP2, 15 mM for D187L mutant and 10 mM for L190G mutant.

^b^ – as described in [20].

The effect of L190G substitution on AMP inhibition was striking. The inhibition became biphasic, with the first half maximal inhibition constant (IC_50_) of 84.4±6.73 μM which is more than 140-fold higher than that of the WT FBP2. The IC_50_ for the second phase of the inhibition was 23.3±2.52 mM – about one thousand times higher than physiological concentration of AMP in mammalian tissues [25]. The effect of D187L mutation was less pronounced. The IC_50_ constant for D187L FBP2 was 1.44±0.06 μM, about 2.5-fold higher than that of the WT FBP2 but still about 3.5-times lower than IC_50_ of WT FBP1. However, the maximal observed inhibition of D187L mutant was about 80%, compared to about 95%, which was observed for the WT FBP2. Cooperativity of AMP binding was retained in both mutants, although the second phase of L190G FBP2 inhibition had a lower Hill coefficient (n; 1.68±0.25) as compared to n∼2.0 which was observed for all other FBP forms. These results show that both D187 and L190 residues play role in the mechanism of inhibition of FBP2 by AMP and that the L190 residue was critical for this inhibition.

### Residues D187 and L190 are indispensable for proper quaternary structure and oligomerization of FBP2

**Figure 3:**
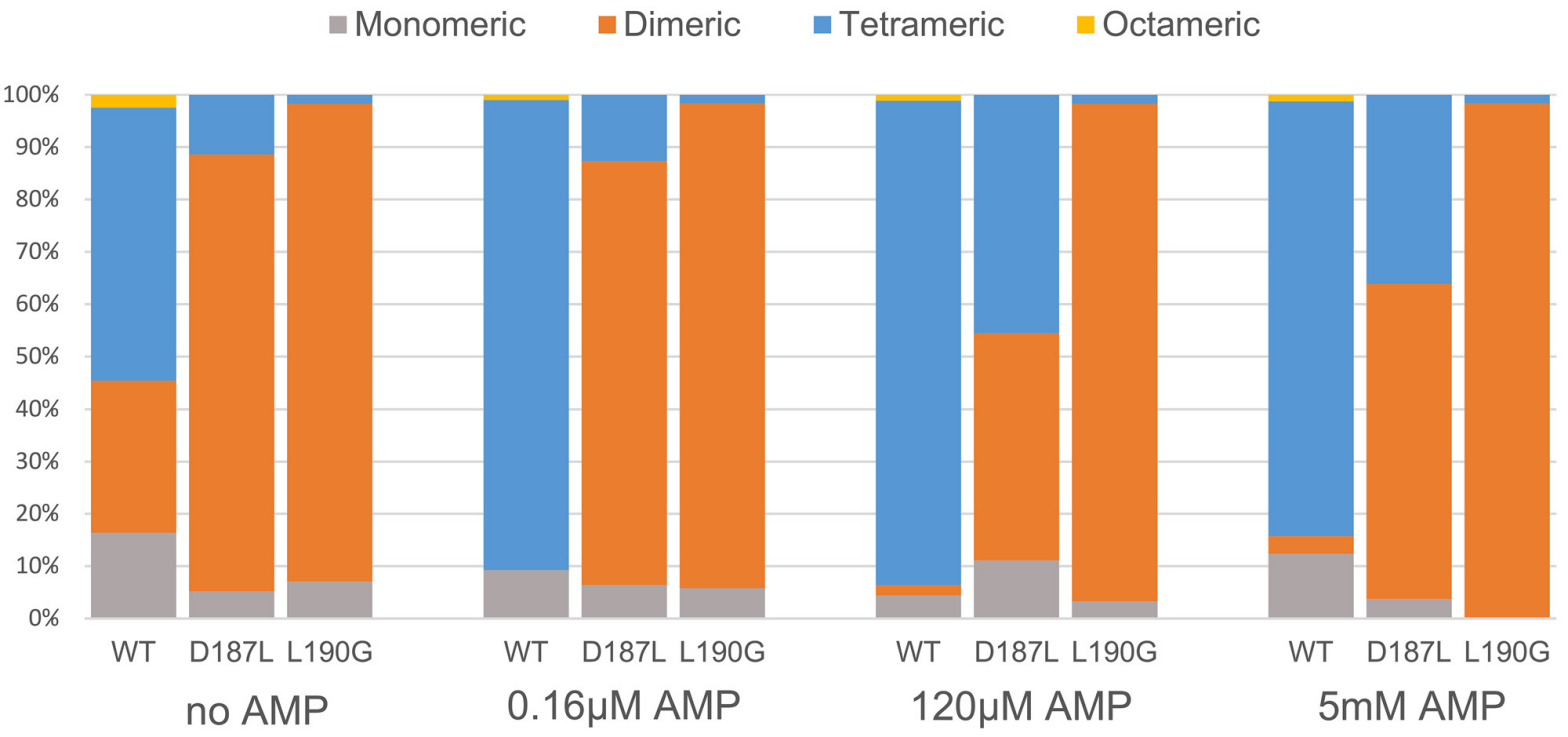
Oligomerization of wild-type and mutant FBP2 in increasing concentrations of AMP Data is presented as percentage of different oligomeric forms in total population of FBP2, determined by analytical centrifugation with interferometric detection.

To test the effect of the introduced mutations on FBP2 quaternary structure, specifically its oligomerization state, we performed sedimentation velocity analytical ultracentrifugation (SV) experiments with the WT, D187L and L190G FBP2 mutants in the presence and absence of AMP. In the initial experiment, we used various concentrations of FBP2 variants: 0.1, 0.5 and 1 mg/ml. As concentration of the proteins had no effect on their oligomerization (see Supplementary Figure 3), the next experiments were performed at 0.5 mg/ml. Figure 3 demonstrates the relative amount of various oligomeric states of the WT FBP2 and the mutants at the different concentrations of AMP. Normalized signal intensity versus sedimentation coefficient graphs and SV experiment data can be found in the supplementary material (Supplementary Figure 3 and Supplementary Table 1).

**Figure 4:**
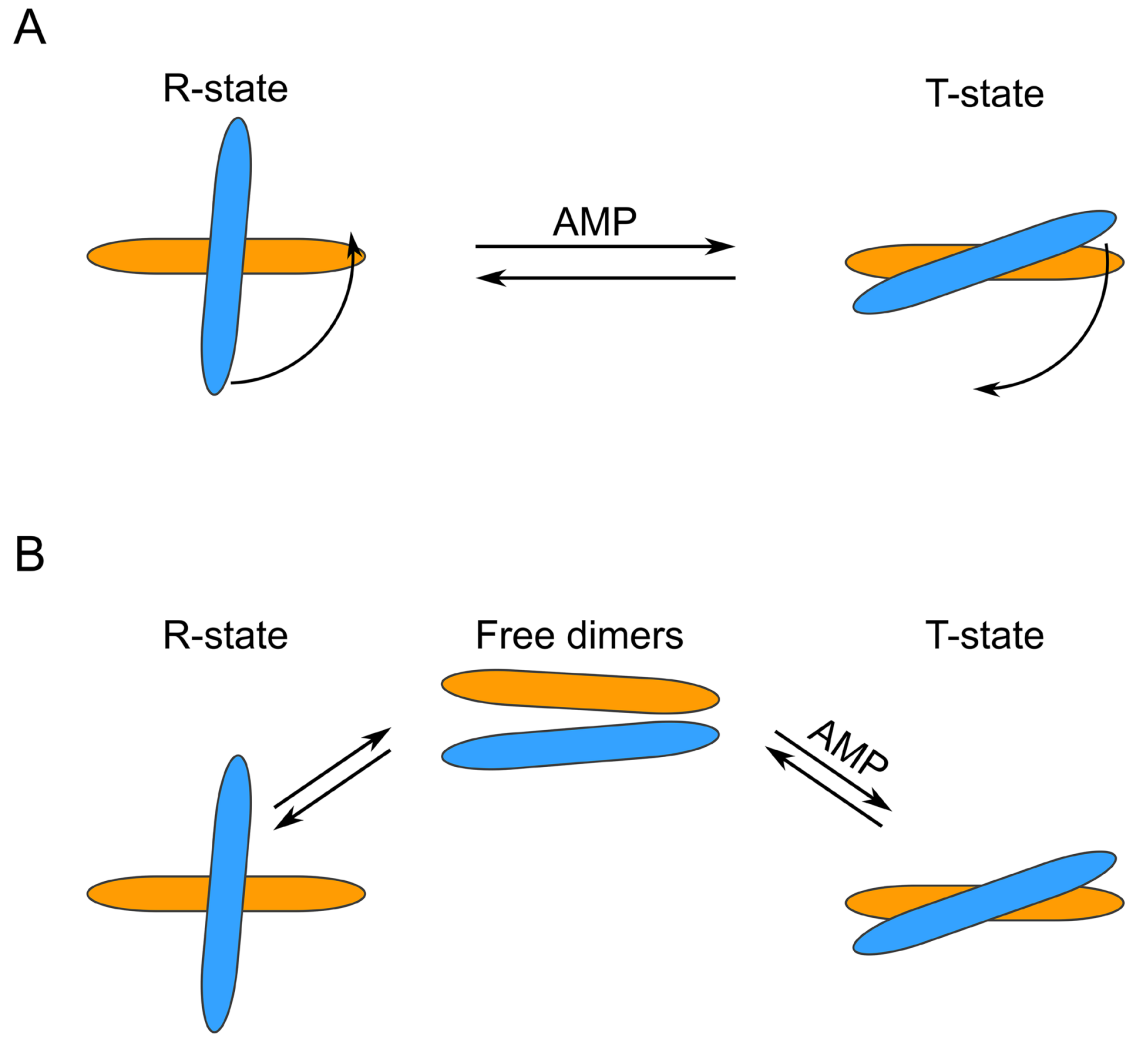
Schematic representation of different FBP2 R- to T-state transition models **(A)** “Dimer rotation” model. **(B)** “Dimer exchange” model. Note that the two models are not mutually exclusive.

To our surprise, we observed that in the absence of AMP, only about half of the WT FBP2 was tetrameric, while about 30% was dimeric and 15% monomeric. The results for FBP2 mutants were even more intriguing. Both mutants existed primarily as dimers – 83.4% for D187L and 91.2% for L190G. Clearly, both D187 and L190 residues are key for stability of the tetrameric form of FBP2. Furthermore, the oligomeric states of both mutants upon addition of AMP differed profoundly from the WT FBP2. At AMP concentration as low as 0.16μM, which is lower than the IC_50_ reported here for the WT FBP2 and lies in the low end of the range of previously reported IC_50_ values [20, 26–27], WT FBP2 was predominantly tetrameric and remained so at higher AMP concentrations (120 μM and 5 mM). However, the mutants proved much more resistant to AMP-induced tetramerization. The L190G FBP2 remained dimeric at all analyzed AMP concentrations (from 0 up to 5 mM). The D187L FBP2 showed no tetramerization at 0.16 μM AMP, but was in about a 45% tetrameric state at 120 μM AMP and 35% tetrameric at 5 mM AMP. This undermines the traditionally held view that FBP is a strictly tetrameric enzyme and confirms the hypothesis based on crystallographic and kinetic studies that residues D187 and L190 are indispensable for the allosteric inhibition by AMP. Both the residues were thought to be crucial for forming interactions between the dimers, so called “leucine lock”, which stabilizes the active cross-like tetrameric form of the enzyme (R-state). However, the mechanism presented here differs significantly from the canonical model which assumes that dissociation of AMP from the flat (“slab-like”) inactive FBP2 tetramer induces rotation towards the active cross-like R-structure [21, 24]. We demonstrate that in the absence of allosteric inhibitors, FBP2 exists in an equilibrium of monomeric, dimeric and tetrameric forms. Remarkably, the dimeric structure of muscle FBP is sufficient to support catalysis: the entirely dimeric L190G FBP2 was fully catalytically-active. AMP and, presumably, NAD^+^, induces tetramerization of the enzyme which then exists in an equilibrium of R and T-state tetramers (NAD^+^ was shown to inhibit FBP2 in the same manner as AMP, however, the IC_50_ was about 250 times higher than in the case of AMP [26]). Contrary to what was assumed so far, the switch between T and R states does not necessarily involve rotation of the upper and lower dimers but may be achieved through their exchange between tetramers. Such continuous assembly and disassembly of FBP2 tetramers would require at least a part of FBP2 molecules to exist as dimers at any given time. This is in agreement with the experimental evidence: WT FBP2 dimers (as well as monomers) were detected even at 5mM concentration of AMP. A similar phenomenon of subunit exchange was reported earlier for FBP1, however, unlike here, it was completely abolished in the presence of AMP [28]. These results make a case for “dimer exchange” rather than “dimer rotation” explanation of the R-to-T-state switch of FBP2. Both models are schematically presented in Figure 4.

FBP from E. coli has also been demonstrated to be dimeric. However, in contrast to the mammalian enzyme, tetramerization activates the E. coli enzyme. Moreover, tetramerization is caused by citrate or phosphoenolpyruvate (PEP) and not AMP. The binding site of citrate and PEP is not conserved between E. coli and mammalian FBP, despite high-level conservation of the overall fold [29]. Clearly, the mechanism described here must have evolved after the separation of mammalian and bacterial lineages.

Based on the kinetic results, we also propose the existence of an additional mechanism of FBP2 inhibition by AMP, different from the classical one which assumes that binding of AMP induces the T-state tetramer formation, where the catalytic loops of 52-72 are stabilized in the inactive conformation [22–23]. Since the L190G FBP2 was inhibited by high concentrations of AMP even though it was almost completely dimeric thus, evidently, AMP caused FBP2 inhibition based only on structural changes within the dimer.

Since the classic AMP binding sites were not affected by the mutations, the observed inhibition of the mutants suggests the presence of the second AMP binding site within the FBP2 subunit. It may be presumed that in high AMP concentration, the inhibitor may associate with the active site and inhibit the activity non-allosterically. The active sites within the dimer may communicate with each other as they are formed by residues belonging to both subunits (residue R243 participates in F-1,6-BP binding in the adjacent monomer within the dimer) [21, 30], and hence the cooperative signal may be transmitted between them. Such mechanism of cooperativity is in line with the results of our study on F-1,6-BP hydrolysis, which demonstrated the cooperativity of the reaction, with n between 1.6 and 2 for all studied FBP2 forms.

### Mutant and WT FBP2 have a similar effect on cell survival in hypoxia and reoxygenation

**Figure 5:**
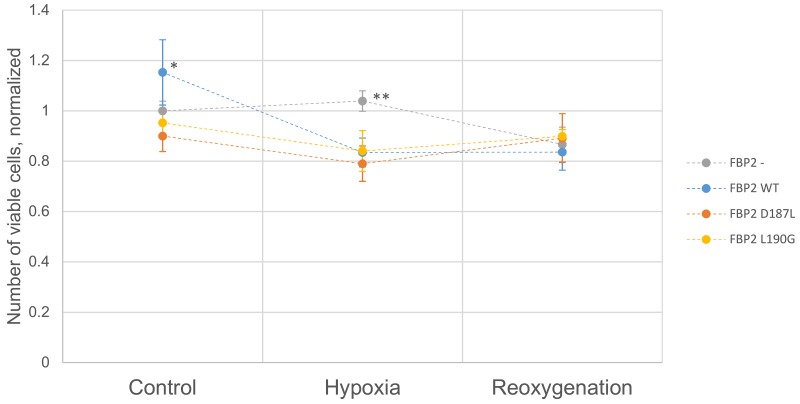
Viability of HL1 FBP2- cells after transfection with FBP2 in hypoxia and reoxygenation Data is presented as mean number of cells normalized to FBP2- cells in control conditions (n=3 culture wells). Error bars represent standard deviation. ^*^ - statistical significance with p-value of 0.033 vs FBP2-, 0.0033 vs FBP2 D187L, and 0.011 vs FBP2 L190G. ^**^ - statistical significance with p-value of 5.7×10^−5^ vs FBP2 WT, 6.8×10^−5^ vs FBP2 D187L, and 8.8×10^−4^ vs FBP2 L190G. p-values were obtained using Student’s *T*-test.

To check the effect of introduced mutations on cell survival, we transfected cardiomyocytes with partially silenced FBP2 expression (the FBP2- cells) with the WT, D187L and L190G FBP2 protein. After overnight incubation with various FBP2 forms, the cells were divided into three groups which were cultured in one of the following conditions: 4 hours of normoxia (control), 4 hours of hypoxia, or 3 hours of hypoxia and subsequently 1 hour of normoxia (reoxygenation experiment). Cells were then harvested and the number of viable cells determined using capillary flow cytometry (Figure 5). In normoxia, the number of cells transfected with WT FBP2 was higher than the number of untransfected FBP2- cells. Differences between cells transfected with both the mutants and untransfected cells were not statistically significant. However, in hypoxia, the number of untransfected FBP2- cells was significantly higher than the number of cells transfected with any form of FBP2. These results are consistent with data presented earlier demonstrating that FBP2 silencing impairs cell growth in normoxia but increases survival in hypoxia. In the reoxygenation experiment, there were no statistically significant differences between any of the groups, however, the number of cells transfected with D187L and L190G was slightly higher than the other two groups and the number of D187L- and L190G-transfected cells in hypoxia.

### Oligomerization state of FBP2 determines its subcellular localization

It has been previously shown that the subcellular localization of FBP2 depends on extracellular signals [1, 6] and the cell cycle phase [7]. In HL-1 cardiomyocytes, FBP2 may be homogeneously distributed in cytoplasm, actively transported to nucleus, or interact with mitochondria. Results of our previous studies revealed that the physiological role of FBP2 in the organelles is not restricted to its catalytic activity. This raises a question whether the subcellular localization, and thus, function of the enzyme depends on its oligomerization state. To test this, first we checked the subcellular distribution of the FBP2 mutants.

**Figure 6:**
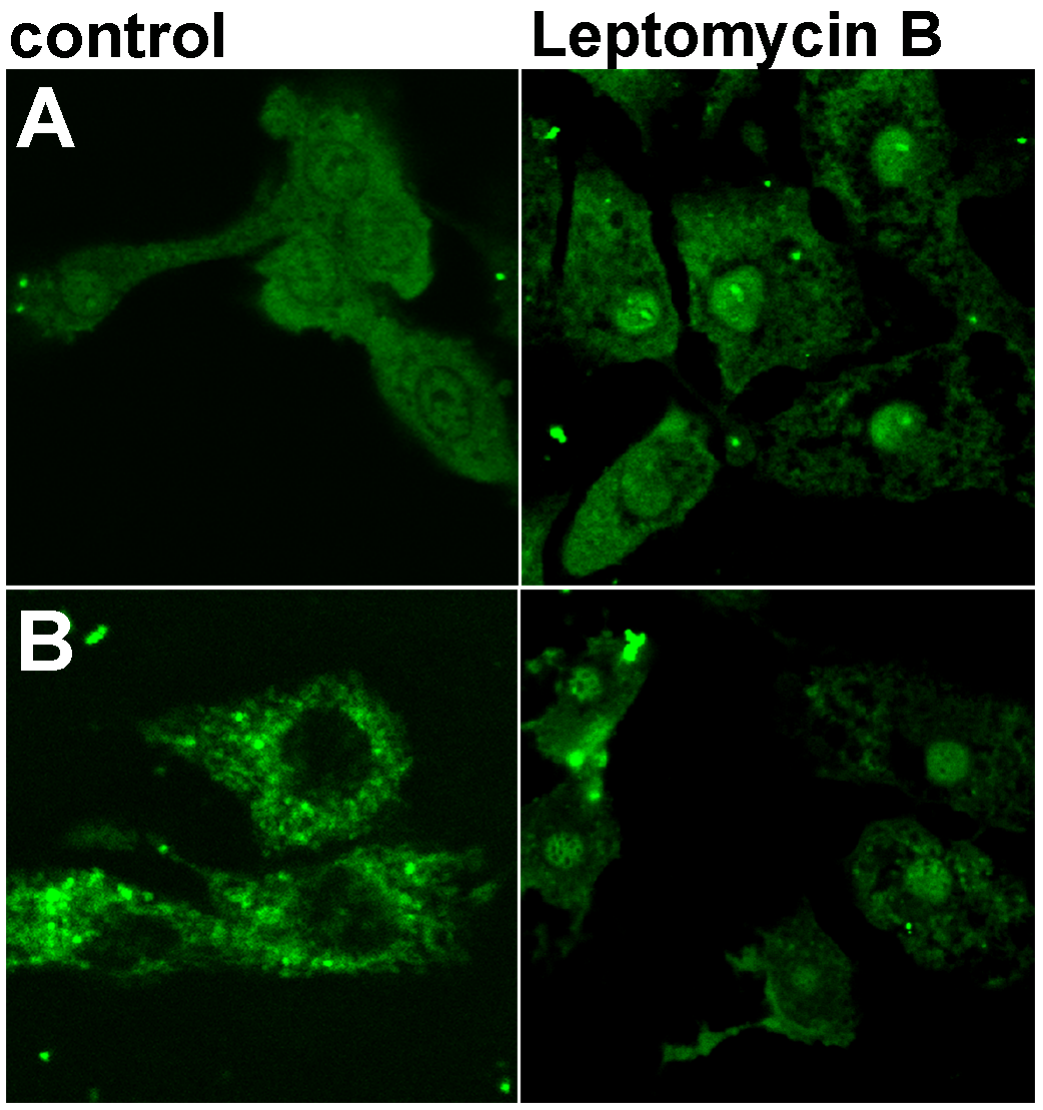
Nuclear localization of WT and L190G FBP2 in control conditions and after the nuclear export blockade with Leptomycin B **(A)** WT FBP2 (predominately tetrameric); **(B)** L190G FBP2 (dimeric form). Bar = 10 μm.

HL-1 cells were transfected with FITC-conjugated WT and mutant FBP2. As a result, nuclear localization of FBP2 was observed in about 50% of HL-1 cells transfected with the WT protein, but only in about 6.6 and 8% of cells incubated with the D187L and L190G mutant, respectively. Moreover, the fluorescent signal from the nuclei of cells transfected with either of the mutants never achieved as high intensity as the signal from nuclei of WT protein transfected cells (Figure 6). Owing to the fact that both the mutants were shown to exist primarily as dimers, it could be assumed that only the tetrameric form of FBP2 was able to pass through the nuclear pore. This, in turn, might suggest that the dimeric and monomeric forms were not recognized by importins. It is known that some proteins (e.g. transcription factor STAT) redistribute from cytoplasm to nucleus following oligomerization because in monomers, NLS of these proteins exists in an inactive state [31]. But in the case of FBP2, it seemed unlikely that the tetramerization process was required for the functional appearance of the NLS [5]. More conceivably, it hindered interactions of FBP2 with nuclear export machinery.

It has been shown that nuclear export of p53 is regulated by the leucine-rich nuclear export signal (NES) located in its tetramerization domain [32]. Although the analysis of FBP2 sequence assigned a low probability of the presence of NES in the protein sequence, we found that the hydrophobic cluster comprised of residues LGEFVL (190-195) may function as a putative NES. In the quaternary structure, these residues are located on the interface of upper and lower dimers, thus they might be inaccessible to CRM1 protein (exportin 1), necessary for the withdrawal of FBP2 from HL-1 cells’ nuclei [6, 21].

To test this hypothesis, we repeated the transfection procedure in the presence of Leptomycin B, an inhibitor of CRM1- and NES-dependent nuclear export. It appeared that FBP2 mutants could be trapped in nuclei by Leptomycin B (Figure 6). This suggests that a prompt export of the dimers was responsible for their mainly cytoplasmic localization, and that tetramerization of FBP2 masks, while dissociation into dimers unmasks the protein’s NES signal peptide.

**Figure 7:**
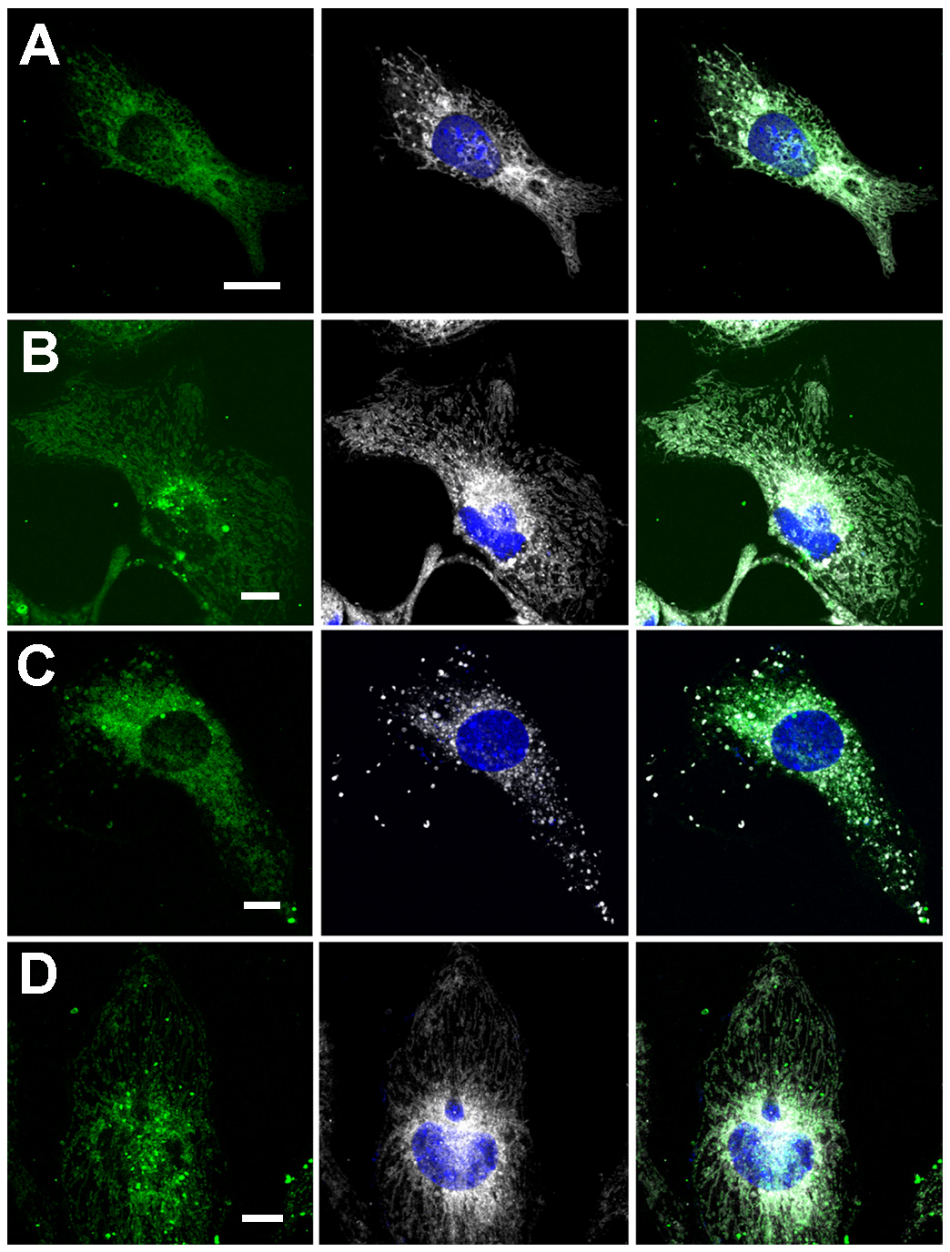
Co-localization of FBP2 forms (green) with mitochondria (gray) in HL-1 cardiomyocytes **(A)** wild-type FBP2 in control conditions; **(B)** L190G mutant in control conditions; **(C)** wild-type FBP2 in the presence of 0.5 mM AMP; **(D)** L190G mutant in the presence of 0.5 mM AMP. Nuclei are counterstained with DAPI. Bar = 5 μm.

In contrast to nucleo-cytoplasmic shuttling, there was no visible difference between the WT FBP2 and the mutants in their capability to interact with mitochondria (Figure 7). This was reflected by the calculated Manders’ overlap coefficient (M1), which was in the range of 0.8 for all the tested proteins. However, increasing concentrations of AMP (up to 500μM) introduced to all of the steps of the transfection procedure led to a drastic reduction of the WT FBP2 potential to interact with the organelles (M1=0.26), without any prominent effect on the D187L and L190G mutants (M1=0.71 and 0.8, respectively). Since the mutants were shown to withstand the AMP-induced tetramerization, it could be inferred that FBP2 binds to mitochondria mainly, if not solely, as a dimer.

**Figure 8:**
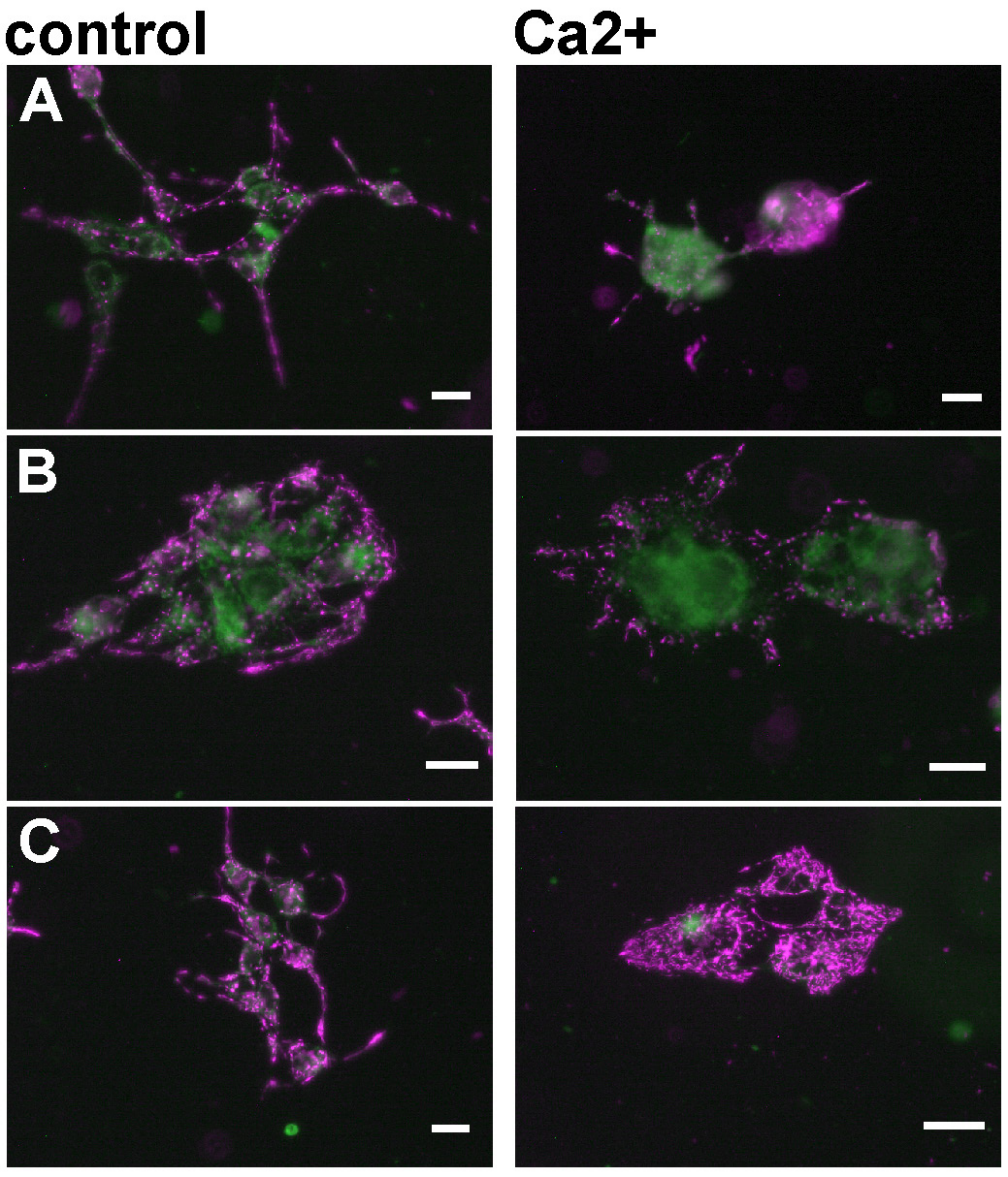
Changes in mitochondrial polarization after transfection of the HL-1 FBP2- cells with different forms of FBP2 in the presence of 0.5 mM AMP **(A)** FBP2- cells, non-transfected; **(B)** FBP2- cells transfected with WT FBP2; **(C)** FBP2- cells transfected with L190G FBP2. Bar = 10 μm.

Previously, we have demonstrated that FBP2 protects mitochondria against high calcium-induced stress [1–2]. Thus, to investigate whether the apparent differences in mitochondria interaction abilities are reflected by disparity in protective potential of the FBP2 WT and mutants, we introduced the proteins into HL-1 FBP2- cells in the presence of AMP and tested susceptibility of the mitochondria to Ca^2+^-induced depolarization. As we have shown before [1], mitochondria of the FBP2- cells were susceptible to depolarization and the cells were prone to detachment from the substrate and clustering (Figure 8A). Transfection of the cells with the FBP2 WT reduced the cells’ tendency to detach (which might be a result of a nuclear action of the protein) under the Ca^2+^ treatment but has no marked effect on mitochondria (Figure 8B). Introduction of D187L and L190G mutants increased mitochondrial polarization (Figure 8C). Collectively, these results show that FBP2 dimers but not tetramers exert the protective effect on mitochondria. It is also worth to note that, in contrast to high Ca^2+^ and H_2_O_2_, hypoxic conditions did not induce FBP2-mitochondria interaction (data not shown).

Our data shed new light on FBP2 quaternary structure and its regulation by small-molecule ligands. We demonstrated that two different FBP2 non-metabolic functions – one connected to the nuclear localization and the other to interaction with mitochondria – are strictly dependent on its oligomerization state and probably mutually exclusive. This discovery was made possible thanks to the utilization of the unique features of the D187L and L190G mutants, the first FBP2 mutants disrupting the oligomerization of the enzyme.

The precise mechanism governing the partition of FBP2 population between its roles in the cytoplasm, nucleus and mitochondria is still unclear. The main factor deciding whether FBP2 activity, both metabolic and non-metabolic, promotes cellular death or survival appears to be the availability of oxygen and different energy sources. In cells growing under normoxic conditions or utilizing non-carbohydrate energy sources, FBP2 is required for growth, survival and cell cycle progression. This is in line with our previous studies [4, 6–7] and studies on metastatic cancer cells [33]. However, under hypoxic conditions, typical for many malignant cancers, FBP2 ceases to play the protective role. In such conditions, silencing of the enzyme promotes cell survival. This agrees with studies showing that in cancer cells, FBP levels are frequently reduced and their overexpression under hypoxia reduces the level of hypoxia-inducible factor-1 α (HIF-1α) protein [9]. HIF-1α is a transcription factor that not only regulates glucose metabolism but also induces angiogenesis and thus, helps cancer cells overcome hypoxic conditions. Therefore, FBP-dependent decrease of HIF-1α inhibits cancer growth, migration and survival [9].

Since in hypoxic conditions, FBP2 does not interact with mitochondria, it could be hypothesized that hypoxia changes the cellular FBP2 dimer-tetramer balance towards tetramer and the tetramer impedes cellular growth and survival by hindering HIF-1α expression. Thus, it appears that modulation of the oligomerization state of FBP2 in a cell by low molecular weight compounds could enable switching of its action from promotion of cell survival to induction of death.

## MATERIALS AND METHODS

### Mutagenesis

Human FBP2 mutants D187L and L190G were created from tag-free WT FBP2 carried on pETite plasmid (Lucigen) with GENEART Site-Directed Mutagenesis kit (Invitrogen) and WALK DNA Polymerase (A&A Biotechnology) using standard protocols provided by the manufacturer. The following primer pairs were used: D187L FW: 5′-GAC CTC TTC ATG CTT CTG CCG GCT CTT GGT GAA-3′, D187L RV: 5′-TTC ACC AAG AGC CGG CAG AAG CAT GAA GAG GTC-3′, L190G FW: 5′-ATG CTT GAC CCG GCT GGT GGT GAA TTT GTC CT-3′, L190G RV: 5′-AGG ACA AAT TCA CCA CCA GCC GGG TCA AGC AT-3′. Sequences were confirmed by DNA sequencing using T7 promoter and T7 terminator primers.

### Protein expression and isolation

All muteins of FBP2 were expressed in *E. coli* and purified according to the following protocol. Clonal colonies of Hi-control BL21(DE3) cells (Lucigen) carrying pETite vectors with inserts encoding tag-free FBP2 were grown on agar-LB (A&A Biotechnology) plates with 30 μg/ml kanamycin (Sigma). Randomly selected clones were used to inoculate 3 ml LB pre-culture and incubated overnight in a shaker incubator set to 37°C, 200 RPM. 500 ml of LB was inoculated with 2 ml of the pre-culture and grown in 37°C, 180 RPM for 5 h. Expression of FBP2 was induced by addition of IPTG (A&A Biotechnology) to a final concentration of 100 μg/ml. Proteins were expressed overnight. Cells were pelleted by centrifugation at 4000xg, 10 min, 4°C. and lysed using BugBuster (MERCK). Cellular debris was removed by centrifugation at 17,000 x g, 25 min, 4°C. Two steps of out-salting were preformed using 50% and 75% saturation of ammonium sulfate. FBP2-containing precipitant was dissolved in 25mM TRIS-HCl buffer, pH 7.5 and dialyzed against the buffer. The protein solution was then incubated with cellulose phosphate (Sigma) at pH 7.1 for 1 h and loaded on 10ml Pierce Centrifuge Columns (ThermoFisher Scientific). The column bed was repeatedly washed with 25 mM TRIS-HCl buffer pH 7.0, 1500 x g, until no absorption peak at 280 nm wavelength could be seen in spectrum of the flowthrough. FBP2 was eluted with 20 mM phosphate buffer, pH 8.0 containing 20 mM fructose-1,6-bisphosphate (F1,6P2, Sigma). Purity of isolated FBP2 was checked using SDS-PAGE.

### Enzyme activity measurement

Measurements of FBP2 activity were conducted at pH 7.5 and 37°C using a glucose 6-phosphate isomerase—glucose 6-phosphate dehydrogenase coupled assay. The reduction of NADP^+^ to NADPH was monitored spectrophotometrically at 340 nm [24]. Parameters were estimated in MATLAB’s curve fitting tool using nonlinear least squares regression with trust-region algorithm and bisquare robust fitting. For assay of FBP2 activation by Mg^2+^ and inhibition by AMP at least triplicate measurement was done for each concentration of a ligand. Data on inhibition by AMP was fitted to the following equations of monophasic (eq. 1) and biphasic (eq. 2) modes of inhibition:

$$\text{v}/{\text{v}}_{\text{max}}=\text{1}-{\text{I}}_{\text{max}}*{\left[\text{I}\right]}^{\text{n}}/\left({\text{IC}}_{50}^{\text{n}}+{\left[\text{I}\right]}^{\text{n}}\right)\;$$ (1)

v/vmax=1−Imax1*[I]n1/(IC50lown1+[I]n1)−Imax2*[I]n2/(IC50highn2+[I]n2) (2)

Where: v – observed reaction rate, v_max_ – reaction rate in the absence of inhibitor, I_max_ – maximal inhibition, [I] – inhibitor concentration, IC_50_ – half-maximal inhibition constant, n – Hill coefficient, I_max1_ – maximal inhibition of the first phase, I_max2_ – maximal inhibition of the second phase, IC_50low_ – half-maximal inhibition constant of the first phase, IC_50high_ – half-maximal inhibition constant of the second phase, n1 – Hill constant of the first phase, n2 – Hill coefficient of the second phase. Parameters of fructose-1,6-bisphosphate hydrolysis were calculated from estimations based on three independent reactions.

### Sedimentation velocity analytical ultracentrifugation

The sedimentation velocity analytical ultra-centrifugation experiments (SV) were conducted in a Beckman Coulter ProteomeLab XL-I ultracentrifuge (software version 6.0, Beckman Coulter Inc., Brea, CA, USA). An-60 Ti rotor and cells with two-channel charcoal filled Epon® centerpieces and sapphire windows were used. The sample sector was filled with 400 μl of solution of FBP2 or its mutant, the reference sector contained 400 μl of appropriate buffer. The protein concentrations were 0.1, 0.5 and 1 mg/ml for proteins without AMP and 0.5 mg/ml for proteins with AMP. The experiment was conducted overnight at 42,000 rpm at 20°C. Sedimentation was monitored by laser interferometry.

Time-corrected [34] scans representing the whole sedimentation process were analyzed using SEDFIT software (http://www.analyticalultracentrifugation.com). The density and dynamic viscosity of the buffer, as well as partial specific volumes of FBP2 variants were estimated using SEDNTERP (http://www.jphilo.mailway.com/download.htm) [35]. For wild type FBP2 and the L190G mutant, sedimentation coefficients (s), frictional ratios (f/f_0_) and apparent molecular weights (MW_app_) were calculated using the continuous c(s) distribution model with 10 points per 1 S. In the case of the D187L mutant, the parameters were calculated using c(s) distribution with bimodal f/f_0_ model. 200 logarithmically distributed sedimentation coefficient values in the range of 1-100 S were fitted to the data. This allowed us to mitigate the influence of high MW aggregates (>15 S) on the analysis. Maximum entropy regularization with p=0.68 was applied [36]. The quality of the fits was assessed using RMSD values, residual distributions and residual histograms.

### Protein labelling

Proteins were dialyzed against borate buffer, pH 9.2 and mixed with FITC (Sigma) dissolved in 50% DMSO to a protein:FITC molar ratio of 1:40. The mixture was incubated in 4°C overnight and then dialyzed against PBS. Protein concentration and number of FITC molecules per FPB2 monomer were then measured spectrophotometrically [5].

### Cell culture

The murine Hl-1 cardiomyocytes, a gift from prof. W.C. Claycomb (Louisiana State University Health Science Center, New Orleans, LA, USA), who first established and characterized the cell line [37], were cultured as described in [6].

A lentiviral cocktail of five shRNAs targeted to mouse FBP2 (Mission shRNA Lentiviral Transduction Particles, Sigma, cat no. NM_007994) was used to down-regulate the gene [1] and the pCMV6-FBP2 plasmid was used to achieve the overexpression of FBP2 in HL-1 cells. The level of FBP2 mRNA was analyzed by PCR as it was described in [7]. The PCR products were subjected to agarose gel electrophoresis with ethidium bromide staining and the density of the gel bands was quantified using the GeneTools v4.0 (Syngene) program.

To roughly assay the amount of FBP2 protein present in homogenates from the HL-1 cells differing in FBP2 expression protein samples were resolved by SDS-PAGE (40 μg of homogenate proteins per lane), with the use of purified FBP2 as the mass marker. FBP2 was detected using Western blotting as described in [1], except that luminol was used as the peroxidase substrate. The antibodies against FBP were tested before on numerous cells and tissues [e.g. 1, 3-4], and proved to be specific against the protein.

### Protein delivery to HL-1 cells and confocal microscopy

FBP2 was delivered into cells using CHARIOT Protein Delivery Reagent (Active Motif). For cytometric analyses, 1.14 μl (2.28 μg) of CHARIOT and 0.3 μg of FBP2 per well in 24-well plates were mixed and applied on cells in the serum-free Claycomb medium according to the manufacturer’s protocol. After 2 h of incubation with the protein delivery mixture, the cells were supplemented with medium containing serum (to the final serum concentration of 6%). No FBP2 was added to control samples. Cells were then cultured overnight. The next day, the protein delivery medium was exchanged with the full culture medium. In experiments with AMP, all the used media were supplemented with AMP to a final concentration of 500 μM. The cells were then cultured as described in the section below.

For fluorescence imaging experiments, cells growing on coverslips in 12-well plates were transfected with 0.5 or 1.5 μg of FITC-conjugated WT FBP2 or the mutants a described above, and grown in the full culture medium for additional 24 h before fixing and staining. The higher amount of FBP2 was shown to ensure relatively fast accumulation of the protein inside the nucleus [5] whereas the lower amount facilitated observation of changes in the FBP2 interaction with mitochondrial network. To visualize mitochondria, cells were stained with 500nM MitoTracker® Deep Red FM (ThermoFisher Scientific) in growth medium for 30 min and then fixed in 4% paraformaldehyde. The coverslips were mounted on microscopic slides in Fluoroshield with DAPI (Sigma).

Images were acquired on FV-1000 confocal microscope (Olympus) with 60x (oil, Plan SApo, NA=1.35) objective. The fluorochromes were excited at 405 (DAPI), 473 (FITC) and 635 (MitoTracker® Deep Red FM) nm and imaged using the Sequential scan option.

To quantify the FBP2-mitochondria co-localization, after subtraction of background from the images, Manders’ overlap coefficient M1 was calculated using the ImageJ 1.47v software and the JACoP plugin [38]. M1 can be concisely defined as the proportion of a signal from the green channel (FBP2-FITC) that overlaps with a signal from the red channel (Mitotracker). Its values can vary from 0 to 1, where the former means no overlap of images and the latter corresponds to 100% co-localization of two images [35]. The non-paired Student’s *t*-test was used for comparisons between two experimental groups.

### Cytometric measurements of HL-1 FBP2- cells viability after hypoxia and reoxygentaion

The HL-1 FBP2- cells, which have the expression of FBP2 reduced to about one-fourth of the normal level, were seeded in 24-well plates at a density of 50, 000 cells/cm^2^. Three wells were used for each delivered protein and growth condition. After overnight culture, cells were transfected as described above. Next, the control plate was incubated in air containing 5% CO_2_ and the two remaining plates in the hypoxic atmosphere of 95% N_2_, 5% CO_2_ in the Brinkubator MIC-101 hypoxic chamber (Billups-rothenberg). After 3 h, one of the plates in hypoxic conditions was transferred to air with 5% CO_2_ with cover removed to improve ventilation, while the other remained in 95% N_2_, 5% CO_2_. All plates were incubated for another hour. Then, cells were thoroughly detached from wells using trypsin, pelleted, and suspended in the culture medium. The cell suspensions were diluted 10- fold in Guava ViaCount reagent (Merck) and incubated in RT for 10 min, following manufacturer’s guidelines. The number and viability of cells were measured using Guava easyCyte flow cytometer (Merck). For each sample two measurements of 1000 events were taken. Events were categorized as viable cells, dead cells, and debris by EasyFit algorithm of GuavaSoft 2.7 ViaCount software. The non-paired Student’s *t*-test was used for comparisons between experimental groups.

### Measurement of mitochondrial polarization and ROS production

Live cells were examined with the Olympus IX71 fluorescence microscope equipped with the Cell^F software (Olympus).

Loss of the mitochondrial membrane potential was detected using JC-1 fluorescent dye (Mitochondrial Permeability Transition Detection Kit, AbD Serotec) according to the manufacturer’s instruction. Polarized mitochondria accumulate more of the dye and are red. In cells with depolarized mitochondria, most of the dye is dispersed in cytoplasm and has green fluorescence. The ratio of red to green fluorescence reflects the degree of the mitochondrial membrane polarization. The JC-1 dye was excited at 488 nm for 500 ms and the emission was observed using a long pass filter, which allowed for simultaneous observation of green (monomers) and red (aggregates) fluorescence. For better discrimination between JC1 aggregates and monomers, the red fluorescence of the aggregates was presented as magenta in the pictures.

To measure intracellular ROS production the cells were loaded with 5 μM dihydrofluorescein diacetate (DHF; 20 min, 37°C), thoroughly rinsed with Hank’s Balanced Salt Solution, and mounted on slides. The fluorescence of the dye was excited at 488 nm for 500 ms.

### MTT assay

The HL-1 cells were seeded into 96-well plates (2×10^3^ cells/well) and cultured for 24 and 96 h. Then the MTT assay was performed as described [39]. To test the effect of the oxidative stress and hypoxic conditions, the cells were treated with 0.25 mM H_2_O_2_ for 2 h (a fresh portion of H_2_O_2_ was added after 1 h of incubation) or were incubated in the hypoxic chamber as described above, before the MTT assay.

The absorbance was measured at 560 nm with a reference filter of 670 nm using a plate reader ASYS UVM340 (Biogenet). For the cells with different expression of FBP2, the number of respective viable cells obtained after 24 h culture in control conditions was assumed to be 1. In all experimental conditions, measurements were averaged from at least eight wells.

## SUPPLEMENTARY MATERIALS FIGURES AND TABLES





## Abbreviations

FBP2: muscle fructose-1,6-bisphosphatase
FBP1: liver fructose-1,6-bisphosphatase
F-1,6-BP: fructose-1,6-bisphosphate
