# Supplementary material for: Dimeric and tetrameric forms of muscle fructose-1,6-bisphosphatase play different roles in the cell
Source: Oncotarget. 2017 Dec 15;8(70):115420–33. doi: 10.18632/oncotarget.23271 (PMC5777782; doi:10.18632/oncotarget.23271)
Supplement: Supplementary file 1 [file oncotarget-08-115420-s001.pdf]

Dimeric and tetrameric forms of muscle fructose-1,6-bisphosphatase play different roles in the cell

SUPPLEMENTARY MATERIALS

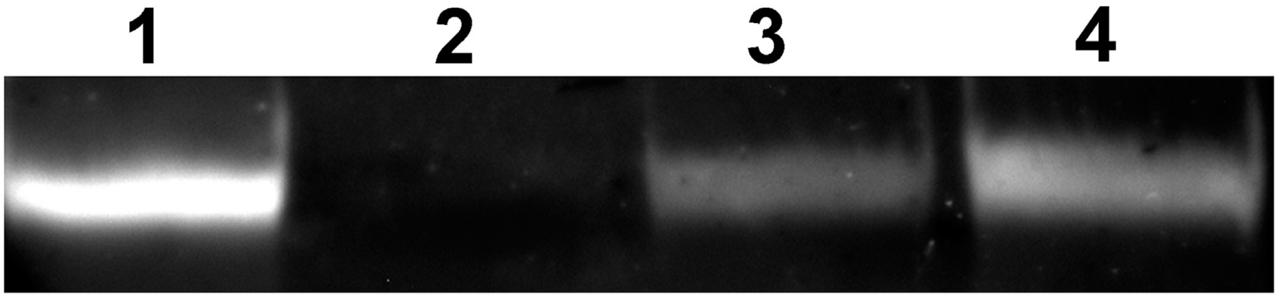

Supplementary Figure 1: Immunoblot analysis of homogenates of HL-1 cells differing in the level of FBP2 expression. Lane 1 – 50 ng of purified FBP2 (control); Lanes 2-4 – homogenates of HL-1 FBP2- (2), WT (3) and FBP2+ (4) cells. The ratio of densities of the protein bands was 0.24 (FBP2-) : 1 (WT) : 2.1 (FBP2+).

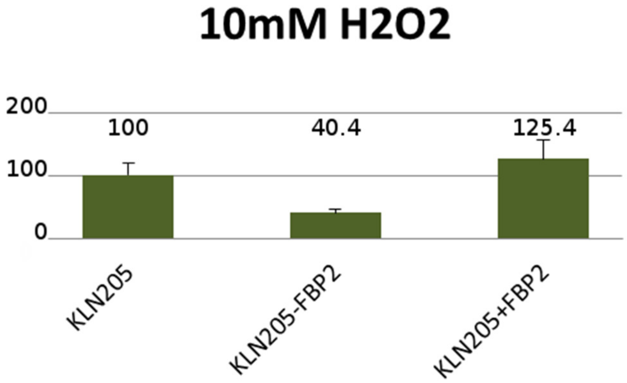

Supplementary Figure 2: Percentage of viable KLN-205 cancer cells expressing different levels of FBP2 in oxidative stress. The cells were cultured as described in Gizak et al. Oncotarget 2015; 6(19):17237-50. KLN205 – control group, KLN205-FBP2 – cells with silenced FBP2 expression, KLN205+FBP2 – cells overexpressing FBP2.

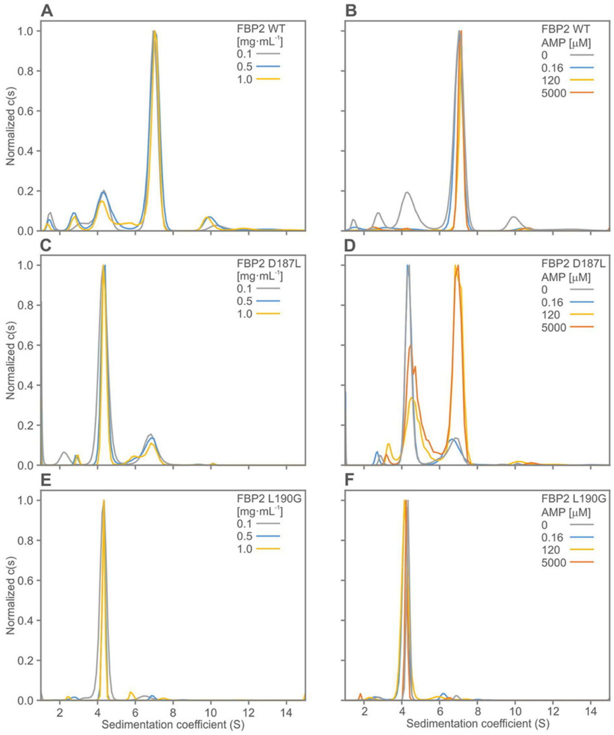

Supplementary Figure 3: Influence of AMP on the oligomerization state of human FBP2 and its mutants. c(s) distributions of **(A, B)** wild type FBP2, **(C, D)** D187L mutant, **(E, F)** L190G mutant calculated using SEDFIT from the interferometric sedimentation velocity data recorded in the absence (A, C, E) and in the presence of AMP (B, D, F). The sedimentation velocity experiment was conducted overnight at 42,000 rpm, 20°C. Scans corresponding to the whole sedimentation process were selected. Each distribution was normalized to the tallest c(s) peak.

Supplementary Table 1: Results derived from the sedimentation velocity analytical ultracentrifugation (SV) data.

See Supplementary File 1
