# Supplementary material for: Dimeric and tetrameric forms of muscle fructose-1,6-bisphosphatase play different roles in the cell
Source: Oncotarget. 2017 Dec 15;8(70):115420–33. doi: 10.18632/oncotarget.23271 (PMC5777782; doi:10.18632/oncotarget.23271)
Supplement: Supplementary file 2 [file oncotarget-08-115420-s002.docx]

**Supplementary Table 1: Results derived from the sedimentation velocity analytical ultracentrifugation (SV) data.**

| Sample | MW_app_ | n | % of the signal | s [S] | f/f_0_ | RMSD |
| --- | --- | --- | --- | --- | --- | --- |
| WT 0.1 mg/ml | 38912 | 1 | 3.7 | 3.12 | 1.24 | 0.002114 |
|  | 62349 | 2 | 20.0 | 4.27 |  |  |
|  | 130130 | 4 | 68.0 | 6.97 |  |  |
|  | 245207 | 8 | 4.3 | 10.63 |  |  |
| WT 0.5 mg/ml | 35939 | 1 | 5.1 | 2.83 | 1.30 | 0.002608 |
|  | 69684 | 2 | 18.1 | 4.40 |  |  |
|  | 139967 | 4 | 65.0 | 7.00 |  |  |
|  | 243130 | 8 | 6.1 | 10.11 |  |  |
| WT 1 mg/ml | 38459 | 1 | 4.0 | 2.82 | 1.36 | 0.004168 |
|  | 73802 | 2 | 15.0 | 4.35 |  |  |
|  | 113622 | 4 | 67.5 | 5.80 |  |  |
|  | 151059 |  |  | 7.01 |  |  |
|  | 253483 | 8 | 5.7 | 9.90 |  |  |
| WT 0.5 mg/ml + 0.16 μM AMP | 39761 | 1 | 2.3 | 2.88 | 1.36 | 0.003271 |
|  | 151062 | 4 | 89.7 | 7.00 |  |  |
|  | 275537 | 8 | 2.0 | 10.46 |  |  |
| WT 0.5 mg/ml + 120 μM AMP | 32276 | 1 | 1.1 | 2.51 | 1.36 | 0.003449 |
|  | 58498 | 2 | 1.7 | 3.73 |  |  |
|  | 67097 |  |  | 4.09 |  |  |
|  | 115020 | 4 | 93.6 | 5.86 |  |  |
|  | 154234 |  |  | 7.13 |  |  |
|  | 276505 | 8 | 2.3 | 10.52 |  |  |
| WT 0.5 mg/ml + 5000 μM AMP | 33645 | 1 | 3.4 | 2.62 | 1.34 | 0.003123 |
|  | 67885 | 2 | 1.9 | 4.18 |  |  |
|  | 150727 | 4 | 91.7 | 7.11 |  |  |
| L190G 0.1 mg/ml | 74357 | 2 | 96.2 | 4.28 | 1.39 | 0.002550 |
|  | 139238 | 4 | 3.6 | 6.51 |  |  |
| L190G 0.5 mg/ml | 36337 | 1 | 3.5 | 2.68 | 1.38 | 0.004486 |
|  | 74447 | 2 | 90.8 | 4.33 |  |  |
|  | 150676 | 4 | 3.5 | 6.93 |  |  |
| L190G 1 mg/ml | 30971 | 1 | 2.0 | 2.45 | 1.36 | 0.005909 |
|  | 72545 | 2 | 88.2 | 4.32 |  |  |
|  | 113020 | 4 | 7.8 | 5.80 |  |  |
|  | 166019 |  |  | 7.50 |  |  |
| L190G 0.5 mg/ml + 0.16 μM AMP | 36852 | 1 | 2.9 | 2.67 | 1.40 | 0.003017 |
|  | 71902 | 2 | 92.9 | 4.16 |  |  |
|  | 131118 | 4 | 3.4 | 6.21 |  |  |
| L190G 0.5 mg/ml + 120 μM AMP | 31071 | 1 | 1.6 | 2.37 | 1.41 | 0.003415 |
|  | 72703 | 2 | 93.8 | 4.17 |  |  |
|  | 124775 | 4 | 4.4 | 5.98 |  |  |
|  | 169706 |  |  | 7.34 |  |  |
| L190G 0.5 mg/ml + 5000 μM AMP | 18395 | 1 | 2.4 | 1.83 | 1.31 | 0.004009 |
|  | 66456 | 2 | 93.6 | 4.23 |  |  |
|  | 128622 | 4 | 3.2 | 6.57 |  |  |
| D187L 0.1 mg/ml | 24881 | 1 | 4.9 | 2.24 | 1.28 | 0.003191 |
|  | 66629 | 2 | 77.1 | 4.31 |  |  |
|  | 131714 | 4 | 17.6 | 6.79 |  |  |
| D187L 0.5 mg/ml | 37308 | 1 | 2.3 | 2.89 | 1.29 | 0.003263 |
|  | 69034 | 2 | 74.0 | 4.36 |  |  |
|  | 133663 | 4 | 20.3 | 6.78 |  |  |
| D187L 1 mg/ml | 37856 | 1 | 2.4 | 2.96 | 1.28 | 0.003861 |
|  | 67840 | 2 | 72.1 | 4.36 |  |  |
|  | 134283 | 4 | 17.2 | 6.87 |  |  |
| D187L 0.5 mg/ml + 0.16 μM AMP | 33264 | 1 | 2.9 | 2.70 | 1.28 | 0.003085 |
|  | 68449 | 2 | 73.8 | 4.37 |  |  |
|  | 129951 | 4 | 23.2 | 6.69 |  |  |
| D187L 0.5 mg/ml + 120 μM AMP | 42956 | 1 | 4.0 | 3.62 | 1.13 | 0.005768 |
|  | 61928 | 2 | 31.3 | 4.63 |  |  |
|  | 113739 | 4 | 65.8 | 6.94 |  |  |
| D187L 0.5 mg/ml + 5000 μM AMP | 36382 | 1 | 1.4 | 3.25 | 1.13 | 0.005789 |
|  | 63800 | 2 | 45.1 | 4.72 |  |  |
|  | 112543 | 4 | 54.3 | 6.90 |  |  |
| MW_app_ = apparent molecular weight; n = number of subunits per oligomer = MW of the proteins calculated from the sequences: approximately 36.6 kDa; s = sedimentation coefficient; f/f_0_ = frictional ratio, RMSD = root mean square deviation, which is a measure of quality of the fit. | | | | | | |
